# Nutritional Intervention and Ergogenic Aids in Sport Performance and Recovery

**DOI:** 10.3390/nu17172806

**Published:** 2025-08-29

**Authors:** David Varillas-Delgado

**Affiliations:** 1 Exercise and Sport Science, Faculty of Health Sciences, Universidad Francisco de Vitoria, 28223 Pozuelo, Spain; david.varillas@ufv.es; 2 SPORTNOMICS S.L., 28922 Madrid, Spain

The interaction between nutritional status, ergogenic aids, and athletic performance has long been a central focus in sports science. By integrating findings from five significant studies, this editorial aims to provide a comprehensive perspective on the current landscape of nutritional interventions and their impact on exercise, sports performance, and recovery. However, the practical applicability of most findings in this field remains limited, requiring careful consideration of several factors, especially in nutrition, supplementation strategies, and gene–gene interactions. Future research should prioritize larger sample sizes and include replication cohorts to enhance the reliability and generalizability of associations in sports science. This Special Issue, entitled “*Nutritional Intervention and Ergogenic Aids in Sport Performance and Recovery*”, compiles five recent advances, explores identified gaps in knowledge, and outlines future research directions.

One of the key contributions in this Special Issue is the longitudinal study by Varillas-Delgado [1], which explores the interplay between genetic polymorphisms and iron supplementation in professional football players. Over three competitive seasons, 48 male athletes were genotyped for variants in angiotensin-converting enzyme (*ACE*) I/D (rs4646994), alpha-actinin 3 (*ACTN3*) c.1729C > T (rs1815739), adenosine monophosphate deaminase 1 (*AMPD1*) c.34C > T (rs17602729), muscle-specific creatine kinase (*CKM*) c.*800A > G (rs8111989), homeostatic iron regulator (*HFE*) c.187C > G (rs1799945), and myosin light-chain kinase (*MLCK*) [c.49C > T (rs2700352) and c.37885C > A (rs28497577)] and monitored for biochemical iron markers and performance metrics. The study introduces a Total Genotype Score (TGS) to predict iron supplementation needs, revealing that players with “optimal” genotypes (e.g., *AMPD1* CC, *HFE* GC) required less supplementation and exhibited superior performance. Iron-deficient players receiving supplementation showed significant improvements in hemoglobin and hematocrit but also had lower match participation and reduced high-speed running metrics, underscoring the endurance performance impact of suboptimal iron status [2,3]. These findings align with previous research highlighting the role of *AMPD1* c.1729C > T polymorphism in muscle energy metabolism and fatigue resistance [4,5]. A recent meta-analysis confirmed that the CC genotype is overrepresented in elite endurance and power athletes, while the T allele is associated with reduced myoadenylate deaminase activity and increased post-exercise fatigue [6]. Moreover, polymorphisms in the *HFE* gene, such as c.187C > G, have been linked to altered iron absorption and hepcidin regulation, influencing iron homeostasis and potentially modulating athletic performance [7,8]. Varillas-Delgado’s work reinforces the value of precision nutrition in elite sport, demonstrating that genetic profiling can guide targeted supplementation strategies to optimize iron status, reduce injury risk, and enhance performance. This integrative approach bridges molecular genetics with applied sports science, offering a predictive framework for individualized nutrition in high-performance environments.

In a randomized, double-blind crossover trial, Salem et al. [9] demonstrated in this Special Issue that short-term beetroot juice (BJ) supplementation significantly enhanced high-intensity resistance performance in trained males. Improvements were observed in repetition volume, movement velocity, and muscle oxygenation (SmO_2_), alongside reductions in peak heart rate and delayed-onset muscle soreness (DOMS). These effects were attributed to increased NO bioavailability via the NO_3_^−^–NO_2_^−^–NO pathway, which facilitates vasodilation, oxygen delivery, and autonomic recovery [10,11,12]. Complementing these findings, Ramírez-Munera et al. [13] investigated the chronic effects of a four-week supplementation protocol combining 500 mg of NO_3_^−^ from amaranth extract and 8 g of CM in professional female soccer players. The intervention led to sustained improvements in maximal speed (Vmax), total distance covered, and post-match anaerobic performance. Notably, plasma NO_3_^−^ levels remained elevated 24 h after the final dose, suggesting prolonged systemic exposure and potential intramuscular storage [9,10]. These findings underscore the relevance of NO precursors in supporting recovery during congested training periods. Both studies [9,13] converge on the notion that NO-mediated adaptations—whether acute or chronic—can enhance neuromuscular efficiency, cardiovascular resilience, and recovery kinetics. However, they also raise important questions regarding long-term safety, optimal dosing, and individual variability. For instance, while BJ is rich in antioxidants that may mitigate nitrosative stress, isolated nitrate salts or extracts may lack such protective compounds [14,15]. Moreover, emerging concerns about N-nitroso compound (NOC) formation at high nitrate intakes warrant further investigation, especially in non-athletic populations [16]. From a practical standpoint, these findings advocate for a periodized approach to NO_3_^−^ and CM supplementation, tailored to the athlete’s training phase, sport-specific demands, and recovery needs. Acute BJ intake may be best suited for resistance training blocks, while chronic NO_3_^−^ + CM strategies could support intermittent sports like soccer during pre-season or tournament phases.

Another notable contribution in this Special Issue is the randomized, double-blind, placebo-controlled crossover trial by Rashki et al. [17], which investigated the effects of acute capsaicin supplementation on recovery and performance in collegiate male futsal players. Participants consumed 12 mg of purified capsaicin 45 min before completing an exercise-induced muscle damage (EIMD) protocol consisting of 200 plyometric jumps. Capsaicin significantly reduced delayed-onset muscle soreness (DOMS) across all time points (immediately, 12, 24, and 48 h post-EIMD), improved vertical jump height (VJH), and increased the pressure pain threshold (PPT) while also reducing thigh circumference (TCM), a marker of muscle swelling. These effects are attributed to capsaicin’s activation of the transient receptor potential vanilloid 1 (TRPV1) channel, which modulates pain perception, reduces pro-inflammatory cytokines (e.g., IL-6, TNF-α), and enhances mitochondrial biogenesis and nitric oxide (NO) bioavailability [18]. TRPV1 activation has been shown to increase intracellular calcium levels, promoting ATP production and improving muscle contractility [19]. Moreover, capsaicin’s analgesic effects are mediated through desensitization of nociceptors and inhibition of neurogenic inflammation [20]. Although no significant improvements were observed in isometric or isokinetic strength metrics, the findings support capsaicin’s role as a recovery aid in high-intensity sports. These results align with previous studies demonstrating capsaicin’s ergogenic potential through enhanced lipid metabolism, reduced perceived exertion, and improved endurance [21]. Future research should explore chronic supplementation protocols, sex-specific responses, and TRPV1 receptor variability to optimize its application in elite sport.

Additionally, Pengelly et al. [22] present a longitudinal observational study examining the influence of iron status on strength and power performance in elite female Australian Football League Women’s (AFLW) players. Over a 10-week preseason, 30 athletes were categorized as iron-deficient (ID; serum ferritin <40 µg/L) or iron-sufficient (IS; ≥40 µg/L). Strength performance (bench press, squat, hip thrust) was up to 13% lower in ID players at baseline, although differences diminished by week 10. Power and speed metrics (countermovement jump, 10 m sprint, maximal velocity) showed marginal differences between groups. The study highlights the prevalence of iron deficiency in female team sport athletes and its potential impact on neuromuscular performance. These findings are consistent with prior research indicating that iron deficiency impairs aerobic capacity, muscle oxygenation, and recovery [23]. Even in the absence of anemia, low ferritin levels can compromise mitochondrial function and energy metabolism, leading to reduced performance [24]. Moreover, functional iron deficiency—characterized by adequate ferritin but impaired iron utilization—may also compromise performance due to elevated hepcidin levels and reduced iron bioavailability [25]. Hepcidin, a key regulator of iron homeostasis, is upregulated during inflammation and intense training, blocking iron export and limiting erythropoiesis [26]. This mechanism is particularly relevant in athletes undergoing high training loads, where iron sequestration may impair adaptation and recovery. The authors advocate for individualized iron monitoring and supplementation strategies to mitigate performance decrements and support athlete health. These recommendations are supported by recent reviews emphasizing the need for personalized thresholds and longitudinal screening in female athletes [27]. Integrating iron status into performance diagnostics may help prevent subclinical deficiencies and optimize training outcomes.

Current research in sports nutrition is increasingly embracing molecular and personalized approaches to optimize both performance and recovery. The integration of genetic insights, targeted supplementation, and physiological monitoring is shaping a new paradigm in athlete care. As the field evolves, future breakthroughs will depend on refining these strategies and understanding individual variability. This Special Issue reflects a pivotal moment in the journey toward more effective, evidence-based interventions that support athletes holistically.
